# Development and temporal validation of SCRAPS scoring tool to predict poor prognosis in gram negative sepsis patients requiring early intensive care or high dependency unit admission

**DOI:** 10.1186/s12879-026-12697-w

**Published:** 2026-02-03

**Authors:** Asha K. Rajan, Lavanya Swaminathan, Freya F. Dsa, Srushti Kesarkar, Elaine Z. Fernandes, Neeraja Raju, Souvik Chaudhuri, Vishal Shanbhag, Vandana Kalwaje Eshwara, Vijayanarayana Kunhikatta, Girish Thunga

**Affiliations:** 1 Department of Pharmacy Practice, Manipal College of Pharmaceutical Sciences, Manipal Academy of Higher Education, Manipal, Karnataka 576104 India; 2 Department of Microbiology, Kasturba Medical College, Manipal Academy of Higher Education, Manipal, Karnataka India; 3 Department of Critical Care Medicine, Kasturba Medical College, Manipal Academy of Higher Education, Manipal, Karnataka India

**Keywords:** Gram-negative sepsis, Septic shock, Carbapenem resistance, Prediction model, Renal replacement therapy

## Abstract

**Background:**

Gram-negative sepsis (GNS) is associated with high global mortality rates, particularly in intensive care unit (ICU) settings. Despite the availability of various sepsis severity scores, there is a lack of predictive tools tailored for GNS. Our study aimed to develop and temporally validate a novel prediction scoring tool for poor prognosis in GNS patients requiring early ICU or High-dependency unit (HDU) admission.

**Methods:**

This retrospective observational study included 312 adult patients with GNS admitted to the ICU or HDU of a tertiary care hospital from January 2016 to December 2020. Primary outcome of interest was poor prognosis. Candidate variables were grouped into demographics, comorbidities, clinical presentation at admission, laboratory data, resistance pattern, and early complications. Independent predictors of poor prognosis were identified using multivariate logistic and cox regression analysis. A prediction scoring tool was developed and temporally validated on a prospective cohort (October-December 2024). All the analysis was conducted using SPSS version 29.0. The study was meticulously designed, implemented, and documented in compliance with the TRIPOD checklist.

**Results:**

Among 312 patients with GNS in the development cohort, 71.4% patients had poor prognosis. We developed an 8-point predictive score comprising of independent predictors: APACHE II score > 18, SOFA score > 7, CRP > 88 mg/L, carbapenem resistance in the first isolate, septic shock within 24 h and RRT requirement within 48 h of admission. SCRAPS score demonstrated strong predictive performance with an area under the curve of 0.801 in both the development and validation cohorts. SCRAPS score ≥ 5 was associated with increased risk of poor prognosis and significantly lower survival (Kaplan-Meier, log rank test, *p* < 0.001).

**Conclusion:**

The SCRAPS score ≥ 5 is a reliable, practical tool for predicting poor prognosis in GNS patients. Its early application could support timely clinical decision-making, improving patient outcomes in the ICU and HDU settings.

**Clinical trial number:**

Not applicable

**Supplementary Information:**

The online version contains supplementary material available at 10.1186/s12879-026-12697-w.

## Introduction

Sepsis remains a global health crisis and is among the leading causes of mortality worldwide. According to the 2017 World Health Organisation’s report, sepsis affected 49 million individuals globally, resulting in 11 million fatalities. The global mortality rate due to sepsis stood at 26.7%, with an alarming 42% observed among intensive care units (ICU) patients [1]. In India, the Global Burden of Disease 2017 report estimated nearly 11 million cases of sepsis, leading to over 3 million deaths [2]. 

Several factors contribute to the high mortality associated with sepsis, including delayed hospital admissions, increasing antimicrobial resistance, inappropriate use of broad-spectrum antibiotics, lack of antimicrobial stewardship policies, and inadequate infection control practices. The complex interplay of hemodynamic, metabolic, and clinical parameters in sepsis underscores the need to identify reliable prediction factors that can guide clinical decision-making and facilitate timely interventions.

Various predictive scoring systems, such as Mortality in the Emergency Department Sepsis (MEDS), Sequential Organ Failure Assessment (SOFA), quick Sequential Organ Failure Assessment (qSOFA), Acute Physiology and Chronic Health Evaluation (APACHE II) and Modified Early Warning Scores (MEWS), have been developed to assess sepsis severity and aid in risk stratification. While these tools have demonstrated clinical utility, they are often limited by their reliance on laboratory-based parameters, restricted validation across diverse patients populations, and limited discriminatory capacity [3–7]. 

Despite advancements in sepsis management, a significant gap persists in predicting poor prognosis, particularly in patients with Gram-negative sepsis (GNS), a critical condition with severe implications. Identifying high-risk patients could enable the optimization of treatment strategies, potentially reducing mortality rates, improving patient outcomes, and minimizing healthcare costs. In cases complicated by sepsis-induced multiorgan failure, septic shock, or multidrug-resistant bacterial infections, mortality rates can rise to 38% [8]. 

Existing predictive models for GNS have primarily focused on factors such as readmission rates, the need for follow-up blood cultures, and antimicrobial resistance patterns [9–13]. However, there is a notable lack of models incorporating key clinical predictors, such as antimicrobial resistance patterns and treatment strategies, into prediction scoring systems. These factors could serve as valuable indicators of poor prognosis.

While current tools effectively assess sepsis severity, there remains a gap in integrating patient-specific characteristics into predicting models for GNS patients. We hypothesize that a combination of patient-centric factors- including demographic variables (age, gender, comorbidities), clinical presentation (symptoms, vital signs, laboratory findings), antimicrobial resistance patterns, and complications during hospitalization- could serve as reliable indicators of poor prognosis in GNS patients. Addressing these unmet clinical needs, our study aims to develop a novel prediction scoring tool to predict poor outcomes in patients with GNS, thereby aiding in clinical decision-making and improving patient care.

## Methods

This study adheres to the Transparent Reporting of a Multivariable Prediction Model for Individual Prognosis or Diagnosis (TRIPOD) statement. The corresponding TRIPOD checklist is available in Supplementary Table 1.

### Ethical statement

The study was conducted following a predefined protocol approved by the Institutional Ethics Committee [IEC] under approval number IEC:82/2021, ensuring methodological rigor and adherence to ethical standards.

### Study design, setting, and patient selection

**Fig. 1:**
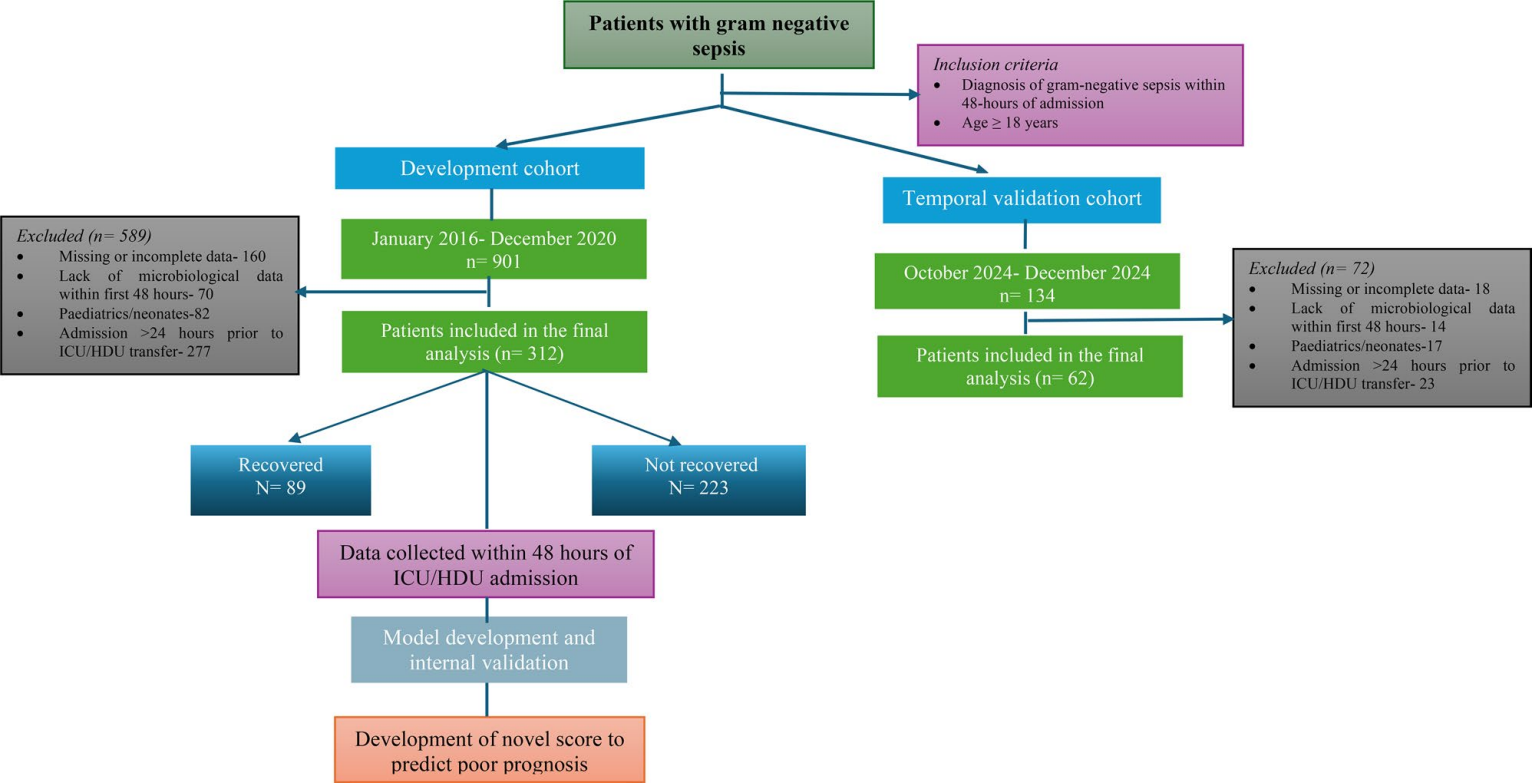
Workflow of model development and temporal internal validation. Note: This validation was performed within the same institution using temporal data from different time periods

This retrospective observational study included all adult patients (≥ 18 years), diagnosed with Gram-negative sepsis (ICD.10 code: A41.5), admitted to the ICU or High Dependency Unit (HDU) within 24 h of admission during the study period. The study was conducted at a tertiary care hospital in Karnataka from January 2016 to December 2020. Exclusion criteria included (i) Missing or incomplete data on primary outcome variables, (ii) Admission > 24 h prior to ICU/HDU transfer (iii) Lack of microbiological data within the first 48 h. A comprehensive dataset was compiled from patient medical records for analysis. Figure 1 illustrates the methodological workflow.

### Definitions

Gram-negative sepsis is a severe, potentially fatal condition characterized by a dysregulated immune response to infection, leading to widespread organ dysfunction. If not promptly managed, it may progress to septic shock, multiple organ failure and death. The condition is specifically caused by aerobic gram-negative microorganisms [14]. Carbapenem resistance in gram-negative organisms is defined as resistance to at least one carbapenem antibiotic (meropenem or/and imipenem) [15]. In this study, carbapenem resistance in the first isolate was defined based on phenotypic susceptibility results obtained from routine microbiological testing. At our institution, standard microbiological workflows allow for reporting susceptibility results within 48 h of specimen collection in most cases.

Septic shock, a subset of sepsis, is distinguished by profound circulatory and metabolic abnormalities that substantially increase mortality risk [16]. Cardiac complications include arrhythmias, atrial fibrillation, and myocardial infarction. Acute kidney injury is defined as a temporary decline in renal function, typically measured by the glomerular filtration rate (GFR), with reduced urine output often serving as an early indicator [17]. ARDS is an acute, diffuse, inflammatory lung condition characterized by impaired oxygenation, pulmonary infiltrates and sudden onset [18]. Encephalopathy refers to a diffuse brain dysfunction, manifesting as coma, acute confusional state, delirium or dementia [19].

#### Outcome measures

The primary outcome of interest was poor prognosis among gram-negative sepsis patients, defined as those who did not improve symptomatically at the end of hospital stay. For analytical purposes, we separately defined in-hospital mortality; where patients died during their hospital stay and discharged against medical advice (DAMA); where patients left the hospital despite recommendations to continue treatment. Patients were classified as “non-recovered” if they either experienced in-hospital mortality or were DAMA due to minimal survival chances, as documented in medical records. Those who showed symptomatic improvement and were discharged in a stable condition were categorized as “recovered”.

The DAMA category predominantly comprised patients with severe multiorgan failure, for whom continuation of intensive care was considered non-beneficial and discharge was initiated by families after counselling. Consequently, these patients closely resembled the in-hospital mortality group in terms of terminal clinical status. Sensitivity analyses excluding or censoring these patients were performed to verify robustness, yielding comparable model performance. Time-to-event was calculated from the time of ICU or HDU admission until the occurrence of the outcome or censoring at hospital discharge or end of follow-up. Patients without the event during the study period were censored at the time of discharge.

### Candidate variables

The candidate variables were selected based on clinical relevance, established scientific evidence, and prior predictive models for GNS. Variables were categorized into demographics, medical history, clinical presentation, laboratory data, resistance patterns and early complications. The timing of variable assessment was standardized based on clinical practice and the availability of data in medical records, as follows:

I. Demographic characteristics and medical history (age, sex comorbidities such as liver disease, type 2 Diabetes Mellitus (T2DM), chronic kidney disease (CKD), Congestive heart failure (CHF), anaemia, malignancy, tuberculosis, AIDS, peripheral vascular disease (PVD), cerebrovascular accident (CVA) and history of alcohol intake) were collected based on patient records at the time of ICU admission.
II. Clinical presentation variables including symptoms such as fever, tachycardia, oliguria, altered sensorium (Glasgow Coma Scale (GCS) < 13) [20], and physical findings like pallor, jaundice (icterus), clubbing and edema were assessed within the first 24 h of ICU admission, as documented in medical charts upon initial evaluation.
III. Laboratory parameters, including albumin, bilirubin, alanine aminotransferase (ALT), aspartate aminotransferase (AST), C-reactive protein, lactate, procalcitonin, haematological markers such as complete blood cell counts, erythrocyte sedimentation rate (ESR). coagulation markers like prothrombin time (PT), international normalised ratio (INR), renal markers such as serum creatinine (Cr), estimated glomerular filtration rate (eGFR) computed using the MDRD-4 variable equation [21], and creatinine clearance (CrCL) determined using the Cockcroft-gault formula at specified intervals [22], were collected as per the first recorded values within 48 h of ICU admission, reflecting the early clinical status and disease severity. The initial values were used to ensure the inclusion of relevant clinical deterioration, consistent with ICU monitoring practices.
IV. Resistance pattern, specifically carbapenem resistance in the first isolate, was determined based on microbiology reports obtained within the first 48 h of ICU admission.
V. Complications including acute respiratory distress syndrome (ARDS), acute kidney injury (AKI), septic shock, and cardiac events were documented based on events occurring within the first 24–48 h, depending on clinical relevance and standard definitions. Specifically, septic shock and ARDS were assessed within 24 h to capture early organ dysfunction, whereas RRT and encephalopathy, which may develop slightly later, were recorded within 48 h. The inclusion of treatment-dependent variables such as renal replacement therapy within 48 h, was considered, as they are part of the early clinical course and frequently used in ICU decision-making, even though they are not readily available at the time of ICU admission.

This approach was implemented to align with clinical workflows and to ensure that variables reflect early disease severity and guide timely prediction evaluation. The choice of 24- or 48- hour windows was based on clinical relevance, the expected timeline of organ dysfunction and existing ICU monitoring protocols.

### Handling of missing data

Missing data for each variable are presented in supplementary Table 2. Variables with missing values exceeding 20% were excluded from the analysis to ensure data integrity and reliability. While microbiological data such as causative pathogens and antibiotic susceptibility patterns are clinically relevant in sepsis management, these variables were excluded from model development due to a substantial proportion of missing data (~ 30%). As the missingness appeared to be related to data recording processes rather than underlying patient characteristics, we considered the possibility of data being missing at random (MAR). However, the pattern and extent of missing data did not meet criteria for robust multiple imputation.

A detailed list of variables where median imputation was applied is provided in Supplementary Table 2.

### Temporal validation

The temporal validation cohort conducted prospectively included 62 patients from a screened population of 132 admitted between October and December 2024 at the same study centre. The same inclusion and exclusion criteria applied as the development cohort. This cohort was used to assess the temporal stability and performance of the SCRAPS model.

### Statistical analysis

Statistical analysis was conducted using Statistical Package for the Social Sciences (SPSS) version 29.0 (IBM Corp. IBM SPSS Statistics for Windows, Armonk, NY) [23]. Descriptive statistics: Categorical variables were presented as frequencies and percentages. Continuous variables were reported as mean ± standard deviation (SD) or median ± interquartile range (IQR), depending on data distribution (assessed using Kolmogorov-Smirnov and Shapiro-Wilk tests). Multicollinearity analysis: variance inflation factor (VIF) and tolerance (T) values were used to assess multicollinearity. A VIF > 5 and T < 0.1 indicated multicollinearity.

The primary outcome, i.e. poor prognosis at hospital discharge, was treated as a binary outcome for the purposes of model development and validation. Therefore, multivariate logistic regression was used to identify independent predictors. For additional assessment of survival differences over time and for assigning scores, Kaplan-Meier survival analysis and Cox proportional hazards regression were applied as supportive analyses, with time origin defined as the date and time of ICU admission. Censoring was applied at the time of discharge or last follow-up for patients without documented mortality events. All analyses involving time-to-event data, including bootstrap weighting and validation, adhered to this framework by consistently defining the time origin as ICU admission and applying appropriate censoring methods.

#### Development of the new prediction score

A new prediction scoring system was developed following a seven-step statistical framework as described by Bae et al. and Todur P et al. [24, 25]. Scores were assigned based on hazard ratios (HR) derived from Cox-regression analysis. The predictive ability of the new score was validated using a receiver operating characteristic (ROC) curve, with area under the curve (AUC), sensitivity, specificity, p-value, cut-off, and confidence interval determined using Youden’s index. The cut-off level for the new score was analysed using Cox regression to predict poor prognosis.

Continuous variables such as APACHE II, SOFA, and CRP were analyzed in their continuous form initially. Nonlinear relationships were assessed using restricted cubic splines. They were given discrete scores (0,1, and 2) using the tercile method, as previously applied by Oliveria et al. In their approach, the range of each predictor was divided into terciles, allowing for a more nuanced categorization of risk factors [26, 27]. To improve clinical applicability, final cut-offs (APACHE II > 18, SOFA > 7, CRP > 88 mg/L) were selected based on optimal discrimination identified by ROC analysis and supported by prior studies demonstrating similar prognostic thresholds [28–30]. 

For clinical applicability and ease of use, the final SCRAPS score categorized these continuous predictors using pre-specified cut-points derived from widely accepted clinical thresholds and supported by statistical analyses. These cut-points were determined based on optimal discrimination identified through ROC curve analysis and clinical relevance rather than arbitrary grouping.

To assess the robustness of the SCRAPS score, sensitivity analyses was conducted using two approaches with respect to DAMA patients. (a) excluding DAMA patients from the analysis, (b) treating DAMA patients as censored alive at the time of discharge.

Model performance was evaluated by examining both discrimination (C-statistic) and calibration, which was explored through graphical plots as well as calculation of the calibration slope and intercept. Ideally, a perfectly calibrated model would demonstrate a slope of 1 and an intercept of 0 [31]. To capture overall predictive accuracy, we also calculated the Brier score, which reflects contributions from both discrimination and calibration and therefore provides a single measure of overall model performance [32]. For internal validation, we applied bootstrapping with 1000 resamples. This resampling approach is widely recommended, as it quantifies potential overfitting and provides an optimism-adjusted of the model’s performance [33]. 

Finally, we examined the clinical applicability of the model using decision curve analysis (DCA). This method evaluates the net benefit, balancing true positives against false positives, across a range of threshold probabilities. By aligning the analysis with the threshold at which a clinician would choose to intervene, DCA provides insight into the practical value of using the model in real-world decision-making.

#### Validation and clinical utility of the new prediction score

Internal validation was performed using bootstrap resampling (*n* = 1000). Survival analysis was conducted using the Kaplan-Meier plot with the log-rank test. A cutoff score ≥ 5 was used for stratifying survival risk. Temporal validation was performed prospectively over three months within the same institution to assess short-term temporal stability rather than external generalizability. The dataset obtained was assessed for predictive performance of the new score using AUC, sensitivity specificity and odds ratio.

## Results

### Clinical characteristics and patient outcomes in the development and validation cohorts

Among the 901 screened patients with gram genitive sepsis, 312 patients met the inclusion criteria (Fig. 1). The mean age in the development cohort was 56.34 ± 14.84 years, with a predominance of males [211(67.6%)]. The most prevalent comorbidities included type II diabetes mellitus [128(41%)], liver disease [51(16.3%)], history of alcohol intake [38(12.2%), chronic kidney disease [25(8%)], and malignancy [26(8.3%)]. At presentation, altered sensorium [100(32.1%)], and decreased urine output [61(19.6%)] were the most common clinical manifestations, while edema [77(24.7%)] and icterus [62(19.9%)] were the most frequently observed physical signs.

Within the first 24 h of ICU admission, the median APACHE II score was 16 (3–41) and the SOFA score was 6 (1–16). Carbapenem resistance in the first isolate within 48 h was observed in 126 (40.4%) patients. The incidence of complications within 24 h of admission included: 38 (12.2%) patients who developed ARDS, 188 (60.3%) patients presented with AKI, 40 (12.8%) patients had cardiac events, 183 (58.7%) patients had septic shock, and 194 (62.2%) patients had inotrope requirement within 24 h of admission. Additionally, 106 (34%) patients required renal replacement therapy (RRT), and 57 (18.3%) of them developed encephalopathy within 48 h of ICU admission. The primary outcome analysis showed that 71.4% (*n* = 223) patients had a poor prognosis, while 28.5% (*n* = 89) recovered and were discharged. Demographic characteristics, clinical presentations, laboratory variables, and outcomes of the development and validation cohorts is given in Table 1.

### Identifying independent risk factors associated with poor prognosis among GNS patients and development of a prediction scoring tool

The variables that significantly differ between the groups for recovery and non-recovery is given in Table 2. Univariate logistic regression was performed to assess the association between patient characteristics and poor prognosis. Variables with a significance level of *p* < 0.25 were included in the multivariate analysis. Significant predictors of poor prognosis in the multivariate model included: CRP, carbapenem resistance of first isolate, APACHE II on day 1 of ICU stay, shock within 24 h of admission, and RRT requirement within 48 h of admission. (Table 3).

A bootstrap analysis on the multivariable regression model using variables identified with a significance level of *P* < 0.25 from the univariate analysis to predict poor prognosis among GNS patients (*n* = 945 samples), confirmed the significance of APACHE II score, and SOFA score on day 1 of ICU stay, CRP, Carbapenem resistance in first isolate, shock within 24 h of admission, RRT requirement within 48 h of admission (Table 4).

#### Time-to-event and censoring

The median time-to-event was 10 [5-19.75] days. Among the 312 patients included in the analysis, 223 patients experienced the outcome of interest, while 89 patients were censored at hospital discharge without experiencing the event. These details are presented to contextualize the survival analysis and ensure appropriate interpretation of the Cox regression results.

Further multivariate cox regression analysis was conducted using variables with *p* < 0.05 that remained significant after bootstrap analysis. The final predictors of poor prognosis were APACHE II on day 1 of ICU stay, SOFA score on day 1 of ICU stay, CRP, Carbapenem resistance in first isolate, shock within 24 h of admission and RRT requirement within 48 h of admission were significant predictors of poor prognosis (Table 5).

### Predictive performance of APACHE II, SOFA score and CRP

**Fig. 2:**
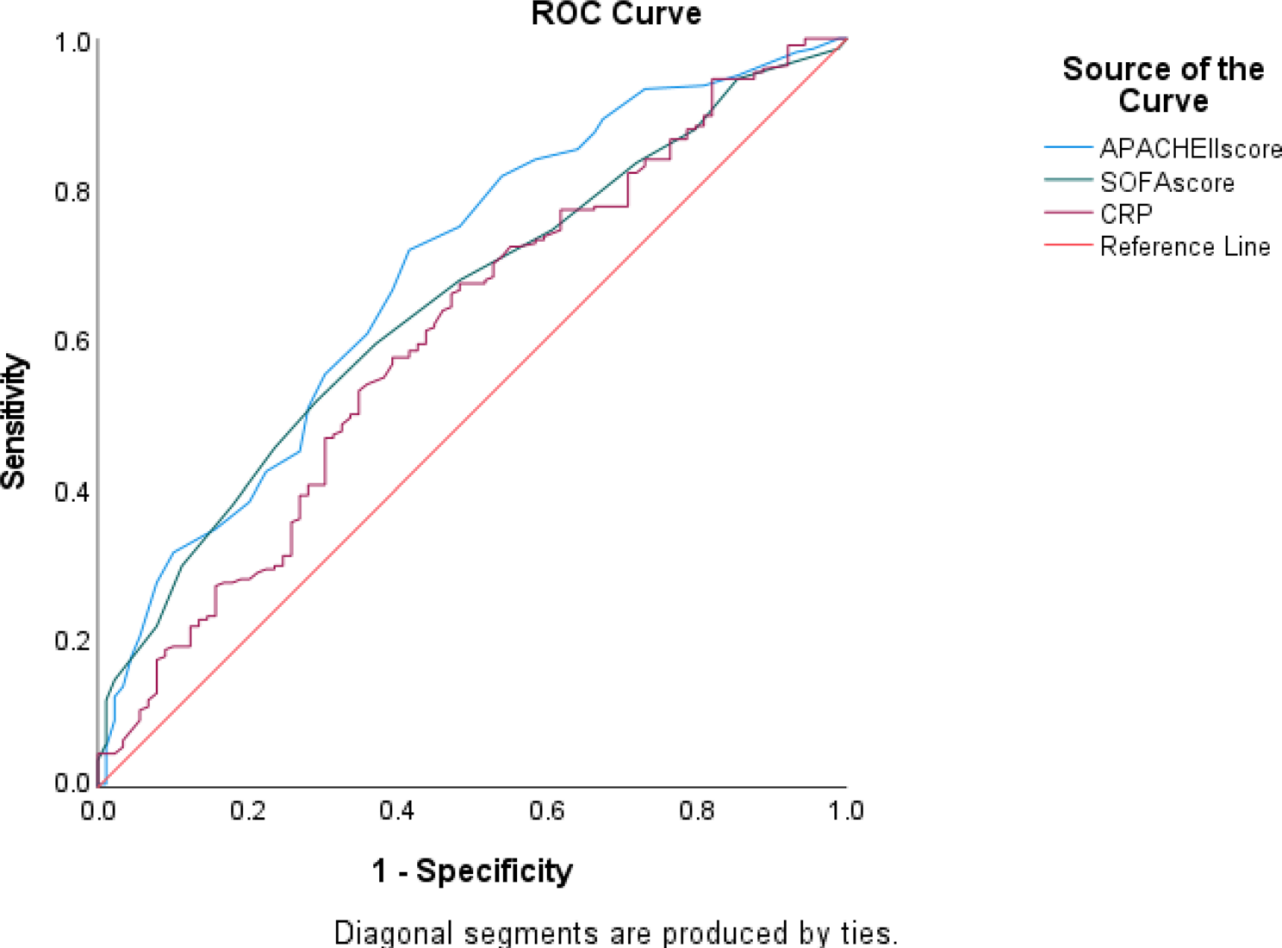
ROC curve of CRP, APACHE II and SOFA score to predict poor prognosis during hospital stay in GNS patients

Receiver operating characteristic (ROC) curve analysis was conducted to assess the discriminative ability of CRP, APACHE II score and SOFA score in predicting poor prognosis. The analysis showed an AUC of 0.686, *P* < 0.00, 95% CI (0.62–0.75), for APACHE II score with 72% sensitivity and 59% specificity. The AUC of SOFA score was 0.645, *P* < 0.00, 95% CI (0.581–0.71) with 68% sensitivity and 52% specificity. The AUC of CRP was 0.604, *P* = 0.004, 95% CI (0.534–0.675) with 66.4% sensitivity and 53% specificity. Notably, the cut-off score of APACHE II score, SOFA score and CRP was noted down at 18, 7, and 88 respectively. (Fig. 2).

A multivariate cox regression analysis incorporating these cut-offs demonstrated that APACHE II, SOFA score and CRP along with other variables, were significant independent predictors of poor prognosis among GNS patients. The analysis showing hazard ratio (HR) for APACHE II score, SOFA score, CRP, carbapenem resistance in first isolate, shock within 24 h of admission, and RRT requirement within 48 h of admission as detailed in Tables 6 and 7.

**Fig. 3:**
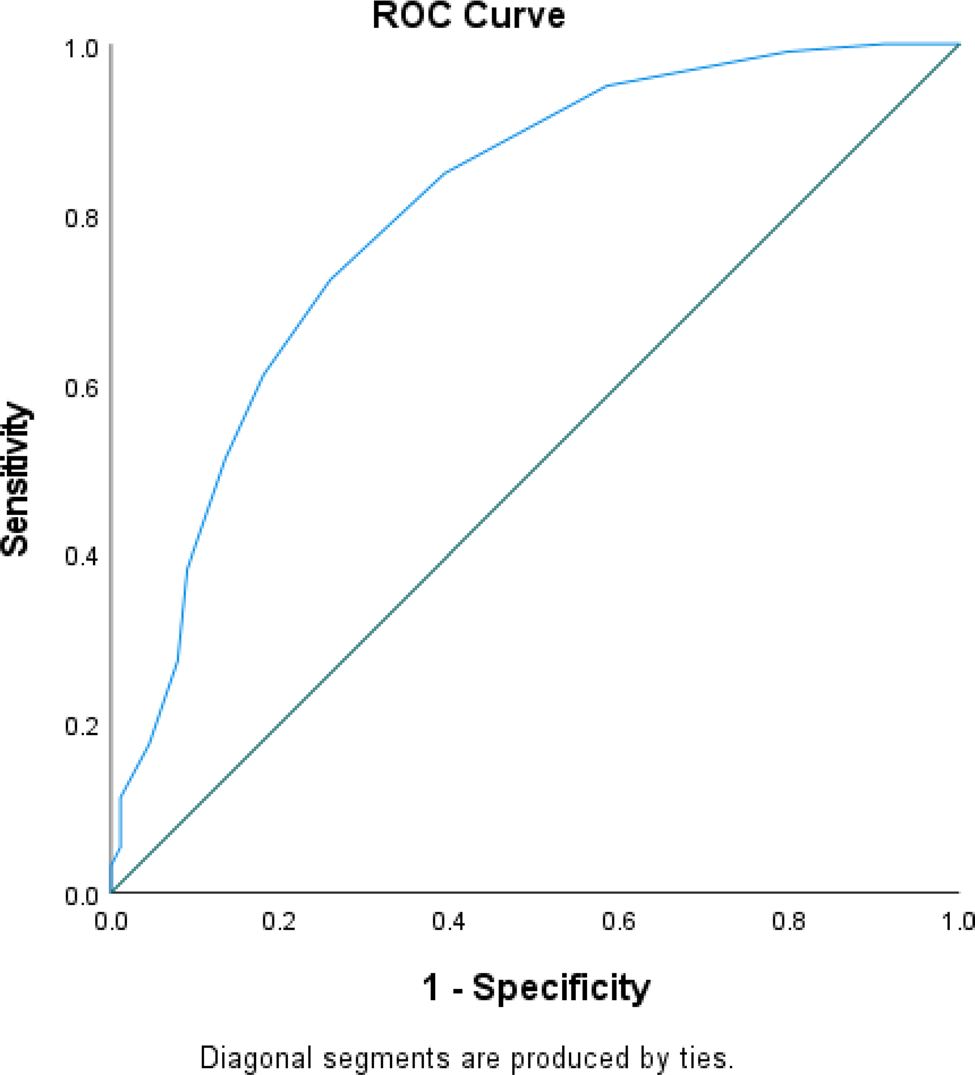
ROC of the SCRAPS score to predict poor prognosis in GNS patients during hospital stay

A new prediction scoring tool was developed, assigning weighted points to each significant predictors such as APACHE II, SOFA score, CRP, carbapenem resistance in first isolate, shock within 24 h of admission, and RRT requirement within 48 h of admission (SCRAPS score). Each predictor was assigned a specific score (1 each for APACHE II score > 18, SOFA score > 7, CRP > 88 and carbapenem resistance in first isolate; 2 each for shock within 24 h of admission and RRT requirement within 48 h of admission), resulting in a maximum score of 8. The highest possible value of the prediction tool was 8. ROC curve was generated to predict poor prognosis among GNS patients. The analysis showed an AUC of 0.801, *P* < 0.00, 95% CI (0.744–0.858). Sensitivity was calculated at 72.2%, while specificity was determined to be 74.2%. Notably, a cut-off score of ≥ 5 was identified (Fig. 3).

### Prediction model development and apparent performance

Six predictors identified through the Cox regression hazard model were subsequently entered into a multivariable logistic regression analysis to construct the prediction model. The model demonstrated a C-statistic of 0.761 (95% CI: 0.745–0.778), indicating good discriminative ability. Calibration analysis showed close agreement between predicted and observed probabilities of poor prognosis, with a calibration slope of 1 and an intercept of 0. The Brier score was 0.167, suggesting reasonable accuracy of predictions.

### Internal validation

To account for optimism, internal validation was carried out using bootstrapping (1000 samples). The optimism-adjusted C-statistic was 0.798 (95% CI: 0.781–0.801). The calibration slope after correction was 0.991 (95% CI: 0.903–0.999), and the intercept was − 0.008 (95% CI: -0.088-0.073). The optimism-corrected brier score was 0.162. Collectively, the calibration slope, intercept, and plots indicated that the model achieved acceptable calibration. Decision-curve analysis further demonstrated that the SCRAPS model yielded a greater net clinical benefit compared with default strategies of treating all patients or none.

### Sensitivity analysis

Sensitivity analysis confirmed the robustness of the SCRAPS score across different outcome definitions. The predictive performance remained consistent when excluding DAMA patients (AUC: 0.768; 95%CI: 0.721–0.797; 70.1%, 73.3%) and when treating them as censored at discharge (AUC: 0.781; 95%CI 0.753–0.791; 72.7%, 75.1%). The results supported the validity of the score, with only minor variations in AUC. These findings reinforce the applicability of the SCRAPS score in varied clinical scenarios.

**Fig. 4:**
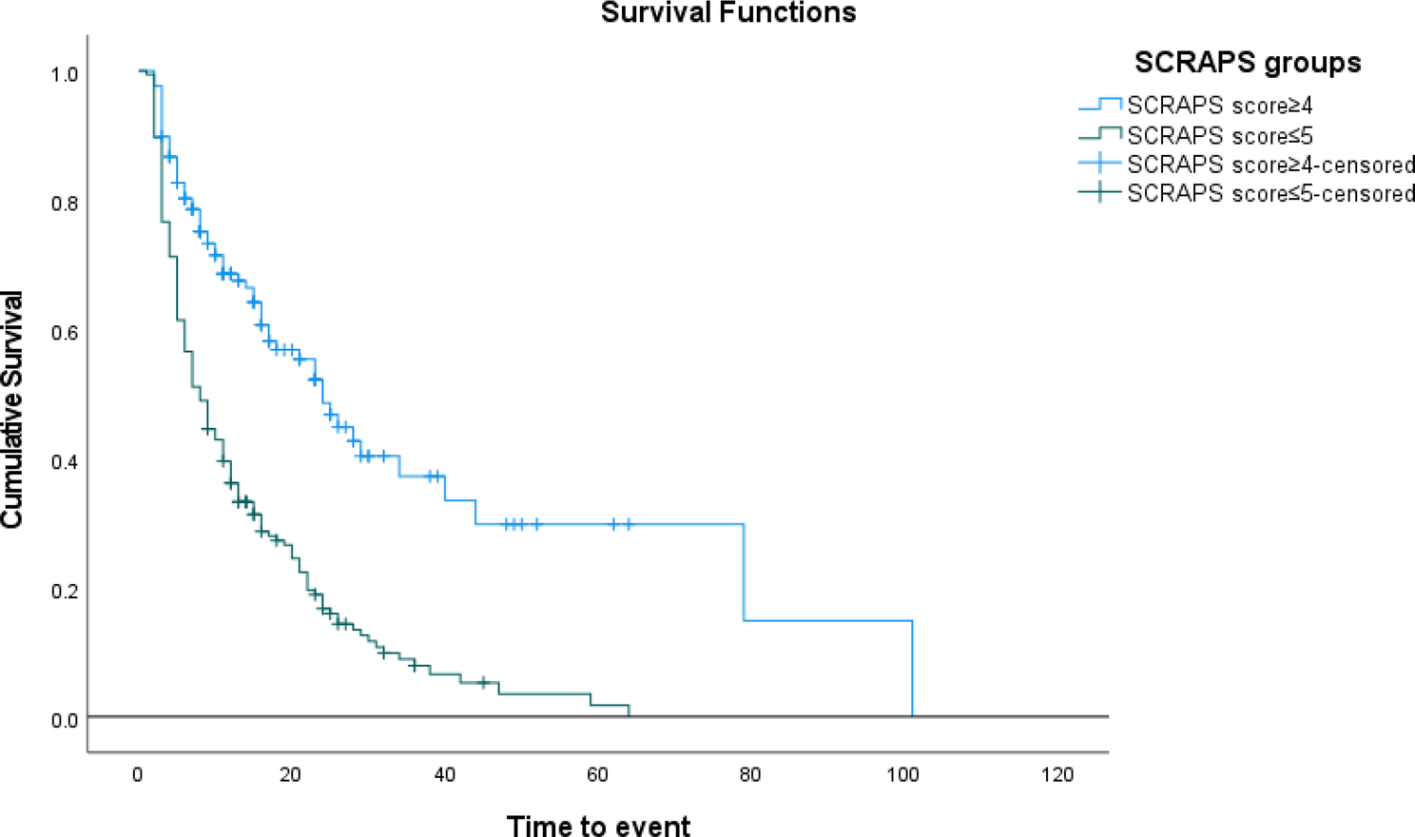
Kaplan-Meier survival curves of GNS patients with the SCRAPS score ≤ 5 and ≥ 4 showed a significant difference, with Log-rank (Mantel-Cox) *P* < 0.001

Kaplan-Meier survival analysis, stratified by the prediction score cut-off ≥ 5, demonstrated a significant difference in survival probability between patients with a prediction score ≥ 5 and those with a score of ≤ 4 (Log rank test *P* < 0.001) (Fig. 4).

### Temporal validation

**Fig. 5:**
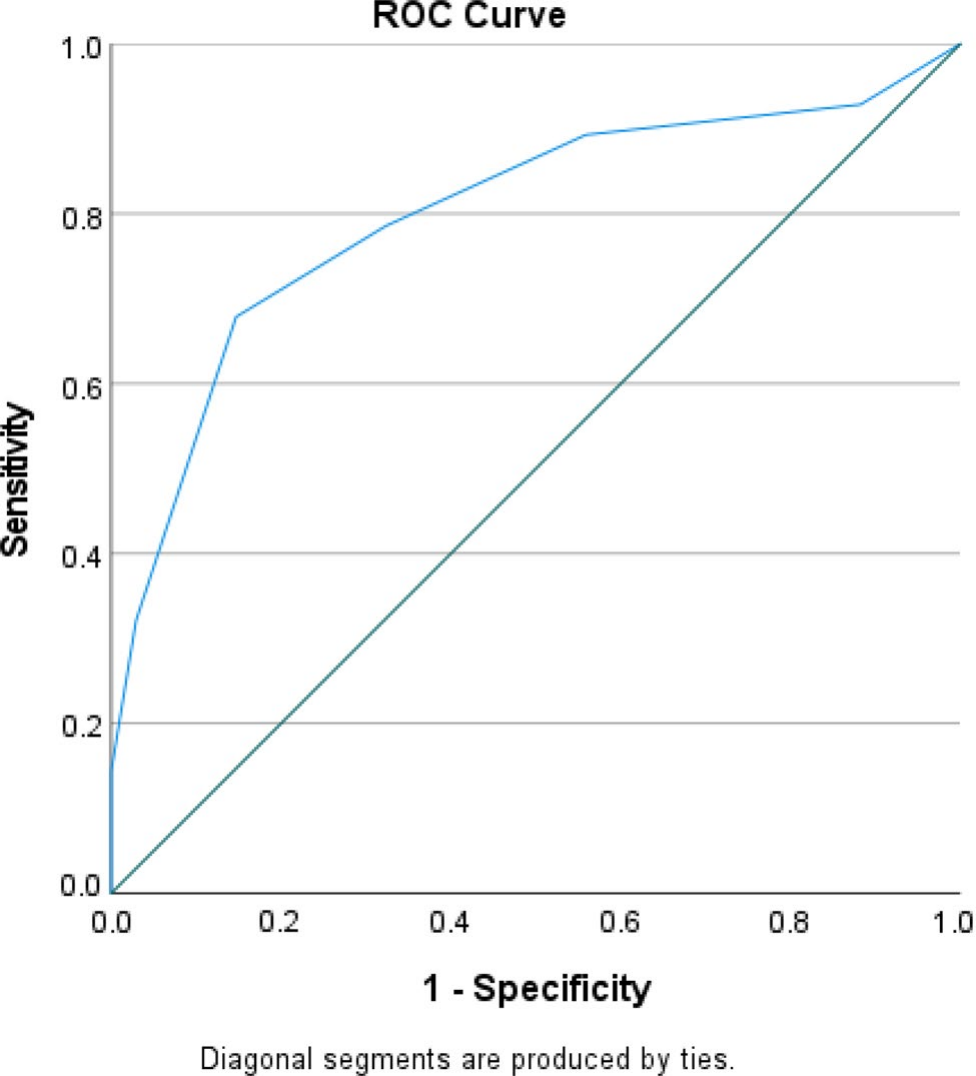
ROC of the SCRAPS score to predict poor prognosis in GNS patients during hospital stay (validation group)

The temporal validation cohort included 62 patients and was used to evaluate the developed scoring tool’s performance. Their baseline characteristics and comorbidities were similar to the development cohort. The ROC curve for the SCRAPS score predicting poor prognosis among GNS patients during hospital stay showed an AUC of 0.801, *P* < 0.00, 95% CI (0.75–0.89) with sensitivity and specificity of 72.2% and 74.2% respectively. (Fig. 5).

### Sample size justification

As this was a retrospective observational study, a formal sample size calculation was not performed prior to data collection. Instead, all eligible patients admitted to the ICU or HDU within 24 h of admission during the study period were included based on the completeness and availability of medical records. To ensure the robustness and stability of the SCRAPS prediction model, the events per variable (EPV) criterion was applied during model development. With 223 events (non-recovered patients) and 39 predictors considered, the EPV was approximately 6. Although this is below the conventional threshold of 10, contemporary evidence supports that models incorporating penalization and internal validation can achieve reliable estimates even at EPV as low as 5–9, provided that candidate selection is clinically guided [34, 35]. We addressed this by carefully selecting predictors grounded in clinical relevance and by applying internal validation techniques such as bootstrap optimism correction to assess and enhance the model’s performance within the limits of the available data, minimizing overfitting risk.

### Clinical example

**Fig. 6:**
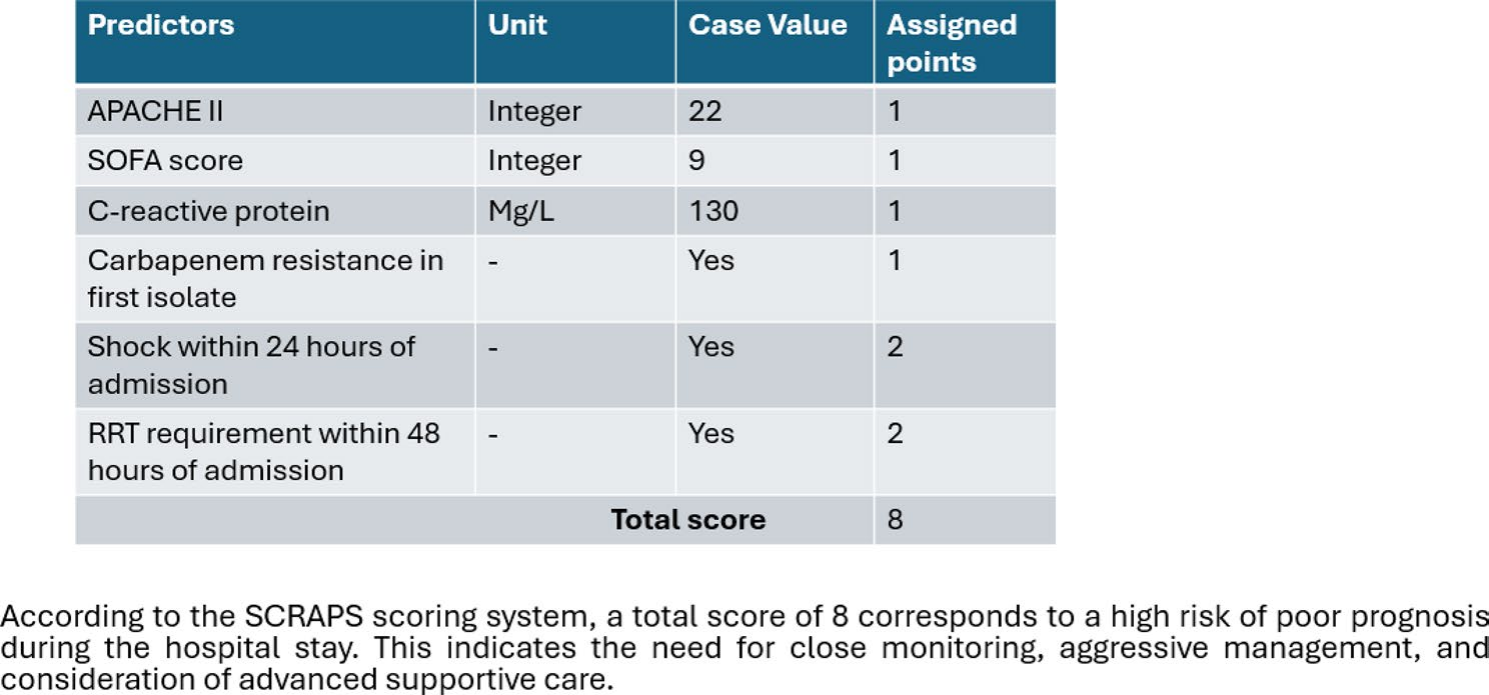
The calculation tool for predicting poor prognosis in gram negative sepsis patients requiring early ICU or HDU admission

Figure 6 shows a clinical example of the application of the calculator on poor prognosis. The predicted poor prognosis of a 65-year-old male patients with gram negative sepsis admitted to the ICU within 24 h of admission, using the clinical parameters available within first 48 h of admission.

## Discussion

Early identification and management of patients at high risk for poor prognosis due to gram-negative sepsis (GNS) is critical for improving clinical outcomes. Despite the limited availability of predictive scoring tools specifically designed for GNS patients, our study addresses this gap by developing and validating a novel prediction scoring tool. This tool incorporates key clinical variables, including resistance patterns, treatment modalities, clinical presentation, and early complications to accurately predict poor prognosis in GNS patients admitted to ICU and HDUs.

Our study identified several key factors significantly associated with poor prognosis. The final predictive model incorporated APACHE II and SOFA scores on day 1 of ICU stay, CRP levels, carbapenem resistance in the first isolate, septic shock within 24 h, and requirement of RRT within 48 h of admission. The scoring tool, which ranges from 0 to 8 points, demonstrated high predictive accuracy, with an AUC of 0.801 and robust sensitivity and specificity in both the development and validation cohorts. A score ≥ 5 was associated with a significantly higher risk of poor prognosis. Early identification of poor prognosis using this tool holds considerable clinical significance. It enables early stratification of high-risk patients, facilitating prompt and targeted therapeutic interventions, which could potentially improve survival and reduce complications.

The APACHE II score > 18 was a significant predictor of poor prognosis in our study. This aligns with prior findings indicating that an APACHE II score ≥ 17 or day 3 of ICU stay is associated with high mortality risk [30]. Some studies have reported cut-offs as low as 15 for predicting ICU mortality, highlighting the variability in cut-off values due to differences in patient populations and timing of score calculation [36]. Our study focused on day 1 scores to enable early risk stratification and intervention. Similarly, a SOFA score > 7 was identified as a strong predictor of poor prognosis, Previous studies have shown that SOFA scores effectively predict ICU mortality and organ dysfunction severity, with higher scores correlating with worse outcomes. While some studies suggest that serial SOFA measurements provide better predictive accuracy, our findings confirm that an early SOFA score on day 1 can provide meaningful prognostic insights [3, 37, 38]. 

CRP > 88 mg/L was also a significant predictor of poor prognosis, consistent with previous studies indicating that elevated CRP levels are associated with increased ICU mortality [39]. CRP’s predictive value extends beyond sepsis, as it reflects systemic inflammation and disease severity. Moreover, the combination of CRP and procalcitonin has been shown to enhance predictive accuracy, supporting the inclusion of CRP in our model [40]. Carbapenem resistance in the first isolate within 48 h of admission was another key predictor. Previous research has established carbapenem resistance as a major challenge in managing gram-negative infections, with resistant strains linked to higher mortality and limited treatment options [41]. A systematic review by Martin A et al. reported that patients with carbapenem-resistant *Enterobacteriaceae* infections face nearly threefold higher risk of mortality compared to those with carbapenem-susceptible infections [42]. Similarly, Jian X et al. highlighted that carbapenem resistance is linked to poorer outcomes in patients with GNB blood stream infections [43]. 

The requirement for RRT within 48 h of admission was a significant predictor of poor prognosis. Previous studies have reported conflicting results regarding early versus delayed RRT initiation in sepsis associated acute kidney injury (SA-AKI) [44]. While some studies suggest that early RRT improves outcomes, others argue that delayed RRT reduces complications and mortality [45]. Septic shock within 24 h of admission was strongly linked to poor prognosis. This finding aligns with previous research showing that septic shock significantly increases mortality risk in sepsis patients [43]. Early identification and aggressive management of septic shock remain critical for improving patient outcomes [46]. 

A key consideration in developing the SCRAPS score was selecting predictors that are available within a clinically actionable window to support early decision-making in critically ill patients with GNS. We recognize that in certain real-world settings, especially where laboratory infrastructure is limited or reporting is delayed, phenotypic susceptibility results may not be available within this timeframe. In such scenarios, alternative or proxy predictors, such as documented prior colonization with resistant organism or risk stratification based on local antibiogram patterns, may be considered to approximate the likelihood of carbapenem resistance.

SCRAPS score is intended for application after the 48 h of ICU or HDU admission, once key laboratory and microbiological data become available. Therefore, it complements rather than replaces early admission-based scores such as APACHE II and SOFA. While discrimination was comparable to these established tools, SCRAPS offers added prediction value by integrating infection-specific variables like carbapenem resistance and early RRT requirement. Clinicians could apply the SCRAPS score at this time point to enhance early management decisions.

Beyond prediction modeling, several predictors incorporated into the SCRAPS score represent potentially modifiable clinical factors. While our study identifies associations, it does not establish causality. Future research should therefore employ causal inference methods to determine whether modifying these exposures could improve outcomes. Approaches such as target trial emulation, marginal structural models, inverse probability weighting, and g-computation provide robust frameworks for estimating causal effects using observational data, particularly in settings where randomized controlled trials are not feasible [47]. Applying such frameworks to gram-negative sepsis could clarify whether early interventions on these modifiable factors directly reduce mortality risk, thereby enhancing the clinical utility of the SCRAPS score and informing evidence-based therapeutic decision-making.

### Strengths and clinical implications

Our study has several strengths. First, the predictive model was developed using routinely collected clinical and laboratory data, making it practical for real-world application without adding to the clinical workload. Second, the model demonstrated its predictability in both the development and temporal validation cohorts. Third, the use of a comprehensive range of clinical variables ensures that the model captures multifaceted nature of GNS and its impact on patient outcomes. The developed tool offers a practical and evidence-based approach for early risk stratification in GNS patients. Its implementation in ICU and HDU settings could support timely clinical decision-making, improve resource allocation and enhance patient care.

### Limitations and future directions

Despite its strengths, our study has certain limitations. First, its retrospective design may introduce spectrum and selection bias, limiting the generalizability of findings to broader ICU populations. Approximately 25% of patients were excluded due to incomplete medical records; although missingness did not show systematic patterns, selection bias remains possible, as excluded patients may have differed in severity, management or outcomes. Second, the study may be underpowered to detect differences in baseline characteristics with small prevalence or modest effect sizes.

Third, subgroup analyses by infection source, pathogen class and resistance status were not feasible due to incomplete records, and microbiological data, including causative pathogens and susceptibility patterns, were excluded from the model, which may have influenced predictive accuracy. Exclusion of microbiological data due to missingness may have limited the model’s ability to capture pathogen-specific risk. It stemmed primarily from unavailable reporting rather than patient-related characteristics, suggesting data were missing at random. Fourth, while the model underwent temporal internal validation within the same institution, the small validation cohort limits generalizability. The similar AUC observed between cohorts likely reflects temporal stability in local clinical practices. Hence, external validation in independent and diverse populations is required to confirm its broader applicability.

We acknowledge that defining poor prognosis as a composite of mortality and DAMA may introduce clinical heterogeneity. However, we included only those DAMA cases that almost exclusively represented terminal events, as confirmed by physician documentation and the sensitivity analyses supported the robustness of this definition. We also recognize the importance of providing user-friendly tools for clinical application. While a web-based calculator is not yet available, we plan to develop one to facilitate bedside implementation. Future studies should employ prospective designs with strategies to reduce missingness (e.g. multiple imputation), validate the model across healthcare environments and explore integration of additional predictive markers such as genomic and inflammatory biomarkers to enhance performance.

## Conclusion

In conclusion, our study developed and validated a novel prediction scoring tool for identifying poor prognosis in patients with gram-negative sepsis. The SCRAPS score, which incorporates APACHE II and SOFA scores, CRP levels, carbapenem resistance, septic shock and early RRT requirement, demonstrated high predictive accuracy and clinical utility. A score ≥ 5 effectively stratified high-risk patients, facilitating early intervention and potentially improving clinical outcomes. The SCRAPS score is intended to complement, rather than replace, existing admission-based tools such as APACHE II and SOFA. It is designed for application after the first 48 h of ICU admission, once the necessary clinical and laboratory parameters are available, to effectively stratify patients at high risk of poor prognosis and facilitate timely intervention. Using this scoring tools at this time point enables more accurate identification of patients at high risk for poor prognosis and supports timely, targeted clinical intervention.

**Table 1:** Clinical characteristics and outcomes of patients in the development and validation groups

Variables	Development group *N* = 312 *n* (%) (Mean ± SD /Median [Range])	Validation group *N* = 62 *n* (%) (Mean ± SD /Median [Range])	*p*-value
Age (years)	56.34 ± 14.84	58.41 ± 10.21	0.065*
Gender (Male)	211 (67.6)	40 (64.5)	0.089**
Tachycardia	44 (14.1)	12 (19.3)	0.756**
Decreased urine output	61 (19.6)	8 (12.9)	0.090**
Altered sensorium	100 (32.1)	9 (14.5)	0.032**
Liver disease	51 (16.3)	5 (8)	0.12**
Type II Diabetes Mellitus	128 (41)	24 (38.7)	0.150**
Chronic kidney disease	25 (8)	6 (9.6)	0.245**
Congestive heart failure	16 (5.1)	4 (6.4)	0.724**
Anemia	4 (1.3)	5 (8)	0.948**
History of alcohol intake	38 (12.2)	11 (17.7)	0.874**
Malignancy	26 (8.3)	4 (6.4)	0.375**
Tuberculosis	7 (2.2)	2 (3.2)	0.324**
AIDS	5 (1.6)	0	0.115**
PVD	14 (4.5)	0	0.875**
CVA	23 (7.4)	1 (1.6)	0.364**
Icterus	62 (19.9)	8 (12.9)	0.025**
Clubbing	5 (1.6)	4 (6.4)	0.745**
Edema	77 (24.7)	14 (22.5)	0.034**
Albumin	3 ± 0.89	2.8 ± 0.56	< 0.001*
Total bilirubin	1.19 [0.15-36]	1.5 [0.24–5.4]	0.034***
AST	45 [7-2742]	38 [16–798]	0.251***
ALT	30 [3-967]	36 [20-1012]	0.752***
C-reactive protein	124.5 [1-511.40]	130 [15.74–376]	0.003***
Prothrombin time	13 [9.6–120]	14 [10.5–120]	< 0.01*
INR	1.21 [0.86–8.96]	0.95 [0.5–5.2]	< 0.01***
ESR	31.5 [1-140]	25 [2–80]	0.874***
Lactate	18 [3-154.80]	16 [136 − 40]	0.072***
Procalcitonin	12.47 [0.47–677.20]	16.40 [0.89-520-47]	0.025***
Carbapenem resistance of first isolate	126 (40.4)	41 (66.1)	0.015**
APACHE II on day 1 of ICU stay	20 [3–43]	23 [6–50]	< 0.001***
SOFA score on day 1 of ICU stay	8 [1–29]	9 [2–30]	< 0.01***
ARDS within 24 h of admission	38 (12.2)	15 (24.1)	0.251**
AKI within 24 h of admission	188 (60.3)	40 (64.5)	< 0.01**
Cardiac events within 24 h of admission	40 (12.8)	16 (25.8)	0.754**
Shock within 24 h of admission	183 (58.7)	28 (45.1)	< 0.01**
Inotrope requirement within 24 h of admission	194 (62.2)	30 (48.3)	0.035**
RRT requirement within 48 h of admission	106 (34)	27 (43.5)	0.047**
Encephalopathy within 48 h of admission	57 (18.3)	24 (38.7)	0.05**

*Independent-T test **Chi-square test ***Mann-Whitney U test, AIDS- acquired immunodeficiency syndrome, PVD- peripheral vascular disease, CVA- cerebrovascular accident, AST- aspartate aminotransferase, ALT- alanine aminotransferase, INR- International normalized ratio, ESR- erythrocyte sedimentation rate, APACHE II- acute physiology and chronic health evaluation, SOFA- sequential organ failure assessment, ARDS- acute respiratory distress syndrome, AKI- acute kidney injury, RRT- renal replacement therapy

**Table 2:** Comparison of baseline characteristics of the development group between recovered and non-recovered patients

Variable	Recovered (*N* = 89) *n* (%) (Mean ± SD/ Median [IQR])	Non-recovered (*N* = 223) n(%) (Mean ± SD/ Median [IQR])	*P* value
Age (years)	54.04 ± 14.17	57.26 ± 15.03	0.084*
Gender (Male)	54 (60.7)	157 (70.4)	0.097**
Tachycardia	11 (12.4)	33 (14.8)	0.576**
Decreased urine output	12 (13.5)	49 (22)	0.088**
Altered sensorium	21 (23.6)	79 (35.4)	0.043**
Liver disease	5 (5.6)	46 (20.6)	0.049**
Type II Diabetes Mellitus	31 (34.8)	97 (43.5)	0.160**
Chronic kidney disease	4 (4.5)	21 (9.4)	0.148**
Congestive heart failure	5 (5.6)	11 (4.9)	0.804**
Anemia	1 (1.1)	3 (1.3)	0.875**
History of alcohol intake	11 (12.4)	27 (12.1)	0.951**
Malignancy	5 (5.6)	21 (9.4)	0.273**
Tuberculosis	1 (1.1)	6 (2.7)	0.399**
AIDS	3 (3.4)	2 (0.9)	0.116**
PVD	4 (4.5)	10 (4.5)	0.997**
CVA	8 (9)	15 (6.7)	0.490**
Icterus	7 (7.9)	55 (24.7)	< 0.001**
Clubbing	1 (1.1)	4 (1.8)	0.670**
Edema	14 (15.7)	63 (28.3)	0.021**
Albumin	3.35 ± 0.99	2.85 ± 0.80	< 0.001*
Total bilirubin	1.03 (0.2-23.63)	1.27 (0.15-36)	0.019***
AST	41 (12–550)	47 (7-2742)	0.200***
ALT	29 (6-295)	31 (3-967)	0.804***
C-reactive protein	86 (1-356)	137 (4.65–511.40)	0.004***
Prothrombin time	12.4 (9.6–84.70)	14 (9.8–120)	< 0.001*
INR	1.13 (0.86–8.96)	1.29 (0.91–8.71)	< 0.001***
ESR	41 (1-140)	30 (1-140)	0.951***
Lactate	18 (3-66.10)	18.2 (4.10-154.80)	0.046***
Procalcitonin	10.34 (0.55–377.50)	13.19 (0.47–677.20)	0.037***
Carbapenem resistance of first isolate	27 (30.3)	99 (44.4)	< 0.001**
APACHE II on day 1 of ICU stay	16 (3–41)	22 (4–43)	< 0.001***
SOFA score on day 1 of ICU stay	6 (1–16)	9 (1–29)	< 0.001***
ARDS within 24 h of admission	7 (7.9)	31 (13.9)	0.141**
AKI within 24 h of admission	38 (42.7)	150 (67.3)	< 0.001**
Cardiac events within 24 h of admission	11 (12.4)	29 (13)	0.878**
Shock within 24 h of admission	20 (22.5)	163 (73.1)	< 0.001**
Inotrope requirement within 24 h of admission	43 (48.3)	151 (67.7)	0.001**
RRT requirement within 48 h of admission	11 (12.4)	95 (42.6)	< 0.001**
Encephalopathy within 48 h of admission	10 (11.2)	47 (21.1)	0.042**

*Independent-T test **Chi-square test ***Mann-Whitney U test, AIDS- acquired immunodeficiency syndrome, PVD- peripheral vascular disease, CVA- cerebrovascular accident, AST- aspartate aminotransferase, ALT- alanine aminotransferase, INR- International normalized ratio, ESR- erythrocyte sedimentation rate, APACHE II- acute physiology and chronic health evaluation, SOFA- sequential organ failure assessment, ARDS- acute respiratory distress syndrome, AKI- acute kidney injury, RRT- renal replacement therapy.

**Table 3:** Univariate and multivariate regression analysis to predict poor prognosis (*n* = 312)

Variable	Univariate analysis		Multivariate analysis	
	*P* value	OR (95% CI)	*P* value	OR (95% CI)
Age	0.085	1.015 (0.99–1.032)		
Gender	0.098	0.649 (0.388–1.084)		
Decreased urine output	0.091	1.807 (0.910–3.588)		
Altered sensorium	0.045	1.776 (1.014–3.113)		
Liver disease	0.006	1.562 (1.134–2.152)		
Diabetes mellitus	0.161	1.440 (0.865–2.399)		
Chronic kidney disease	0.157	2.209 (0.736–6.629)		
Icterus	0.001	3.835 (1.673–8.792)		
Edema	0.022	2.109 (1.111–4.003)		
Albumin	< 0.001	0.530 (0.395–0.711)		
Total bilirubin	0.005	1.123 (1.036–1.218)		
AST	0.126	1.002 (0.999–1.004)		
ALT	0.152	1.003 (0.999–1.008)		
C-reactive protein	0.010	1.003 (1.001–1.005)	0.026	1.003 (1.00-1.006)
INR	< 0.001	2.975 (1.587–5.579)		
Procalcitonin	0.105	1.003 (0.999–1.007)		
PT	< 0.001	1.121 (1.051–1.195)		
Lactate	0.002	1.023 (1.008–1.038)		
Carbapenem resistance of first isolate	0.023	1.833 (1.086–3.094)	0.017	2.161 (1.150–4.060)
APACHE II on day 1 of ICU stay	< 0.001	1.087 (1.052–1.123)	0.041	1.042 (1.002–1.084)
SOFA score on day 1 of ICU stay	< 0.001	1.145 (1.072–1.223)		
ARDS within 24 h of admission	0.146	1.891 (0.800-4.469)		
AKI within 24 h of admission	< 0.001	2.758 (1.665–4.568)		
Shock within 24 h of admission	< 0.001	9.372 (5.252–16.724)	< 0.001	8.156 (4.346–15.307)
Inotrope requirement within 24 h of admission	0.002	2.244 (1.359–3.705)		
RRT requirement within 48 h of admission	< 0.001	5.263 (2.654–10.438)	< 0.001	5.455 (2.505–11.877)
Encephalopathy within 48 h of admission	0.046	2.110 (1.014–4.388)		

**Table 4:** Bootstrap multivariable regression analysis of the variables with *p* < 0.25 in the univariate analysis to predict poor prognosis at the end of hospital stay (*n* = 946 samples)

Variable	Bias	*P* value	BCa 95% CI
APACHE II on day 1 of ICU stay	0.284	0.041	-0.005-0.009
SOFA score on day 1 of ICU stay	-0.057	0.009	-0.308- -0.156
C-reactive protein	0.001	0.016	0.00-0.01
Carbapenem resistance in first isolate	0.165	0.028	-0.213-2.643
Shock within 24 h of admission	0.313	< 0.001	-0.914-1.434
RRT requirement within 48 h of admission	0.284	0.002	0.201–4.117

**Table 5:** Cox-regression analysis to predict poor prognosis (variables used are those which are significant after bootstrap of multivariable regression analysis of 946 samples)

Variable	*P* value	HR (95% CI)
APACHE II on day 1 of ICU stay	0.031	1.340 (1.008–1.68)
SOFA score on day 1 of ICU stay	0.010	1.047 (1.011–1.084)
C-reactive protein	0.023	1.564 (1.112–1850)
Carbapenem resistance in first isolate	0.037	1.23 (1.121–1.532)
Shock within 24 h of admission	< 0.001	2.395 (1.75–3.278)
RRT requirement within 48 h of admission	0.003	1.554 (1.165–2.073)

**Table 6:** Boot strap of Cox-regression analysis after deriving cutoff of APACHE II, SOFA score and CRP to predict poor prognosis (*n* = 1000 samples)

Variables	Bias	*P* value	(BCa 95% CI)	Cutoff as per ROC to predict poor prognosis	Assigned score for new scoring model
APACHE II	0.001	0.024	1.362 (1.008–1.742)	> 18	1
SOFA score	0.00	0.009	1.031 (1.001–1.084)	> 7	1
C-reactive protein	0.00	0.035	1.001 (1.000-1.057)	> 88	1
Carbapenem resistance in first isolate	-0.027	0.043	1.254 (1.103–1.421)	-	1
Shock within 24 h of admission	0.025	0.016	2.294 (1.668–3.155)	-	2
RRT requirement within 48 h of admission	0.00	0.015	1.560 (1.166–2.087)	-	2

**Table 7:** Reporting full model coefficients as per the TRIPOD checklist

Predictor	Coefficient (β)	Standard error	HR (95% CI)	Assigned score
APACHE II	**0.011**	**0.010**	1.362 (1.008–1.742)	1
SOFA score	**0.031**	**0.022**	1.031 (1.001–1.084)	1
C-reactive protein	**0.001**	**0.001**	1.001 (1.000-1.057)	1
Carbapenem resistance in first isolate	**0.305**	**0.141**	1.254 (1.103–1.421)	1
Shock within 24 h of admission	**0.830**	**0.163**	2.294 (1.668–3.155)	2
RRT requirement within 48 h of admission	**0.445**	**0.149**	1.560 (1.166–2.087)	2

## Supplementary Information

Below is the link to the electronic supplementary material.

Supplementary Material 1

Supplementary Material 2

## Data Availability

The datasets used and/or analyzed during the current study are available from the corresponding author at reasonable request. All the other relevant data are within the manuscript and its supplementary information.

## Abbreviations

AKI: Acute kidney injury
APACHE II: Acute physiology and chronic health evaluation
ARDS: Acute respiratory distress syndrome
CKD: Chronic kidney disease
GNS: Gram negative sepsis
ICU: Intensive care unit
MV: Mechanical ventilation
MRSA: Methicillin-resistant Staphylococcus aureus
SOFA: Sequential organ failure assessment
RRT: Renal replacement therapy
