# Supplementary material for: Development and temporal validation of SCRAPS scoring tool to predict poor prognosis in gram negative sepsis patients requiring early intensive care or high dependency unit admission
Source: BMC Infect Dis. 2026 Feb 3;26:487. doi: 10.1186/s12879-026-12697-w (PMC12958660; doi:10.1186/s12879-026-12697-w)

**Supplementary 2: Missing data for included variables in the dataset**

| Variables | Missing, n (%), (n=312) |
| --- | --- |
| C-Reactive Protein (CRP) | 1 (0.3%) |
| Alkaline phosphatase (ALT) | 1 (0.3%) |
| Total bilirubin (TB) | 3 (0.9%) |
| Albumin (Alb) | 2 (0.6%) |

**Supplementary Table 3: Sensitivity analysis**

| Parameters | AUC | P value | 95% CI | Sensitivity | Specificity |
| --- | --- | --- | --- | --- | --- |
| SCRAPS model | 0.801 | <0.00 | 0.744-0.858 | 72.2% | 74.2% |
| Excluding DAMA patients | 0.768 | 0.003 | 0.721-0.797 | 70.1% | 73.3% |
| Treating DAMA patients as censored alive at time of discharge | 0.781 | 0.024 | 0.753-0.791 | 72.7% | 75.1% |

**Supplementary figure 1: Calibration curve**


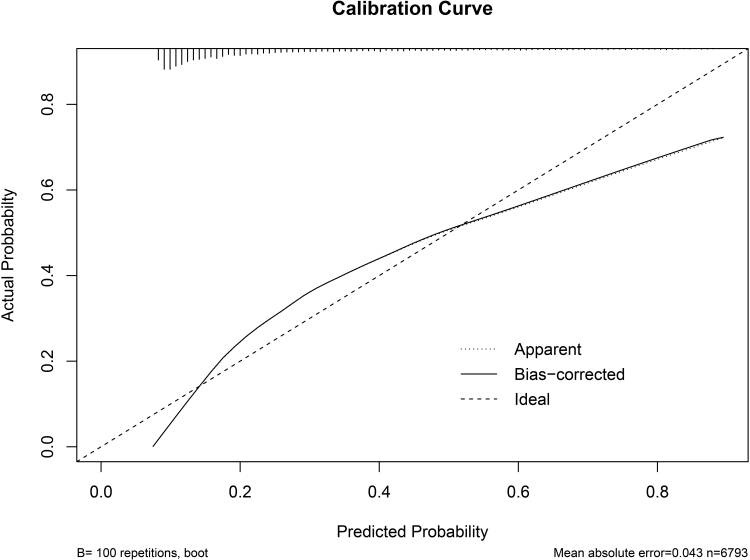


**Supplementary figure 2: Decision curve analysis**


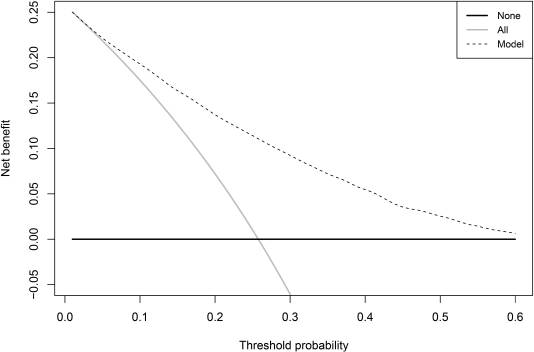

Supplement: Supplementary file 2 — Supplementary Material 2 [file 12879_2026_12697_MOESM2_ESM.docx]
